# A field experiment with elevated atmospheric CO_2_-mediated changes to C_4_ crop-herbivore interactions

**DOI:** 10.1038/srep13923

**Published:** 2015-09-18

**Authors:** Haicui Xie, Kaiqiang Liu, Dandan Sun, Zhenying Wang, Xin Lu, Kanglai He

**Affiliations:** 1 The State Key Laboratory for Biology of Plant Diseases and Insect Pests, Institute of Plant Protection, Chinese Academy of Agricultural Sciences, Beijing 100193, P. R. China; 2 College of Life Science and Technology, Hebei Normal University of Science and Technology, Qinhuangdao 066000, P. R. China; 3 Institute of Plant Protection, Jilin Academy of Agricultural Sciences, Gongzhuling 136100, P. R. China

## Abstract

The effects of elevated CO_2_ (E-CO_2_) on maize and Asian corn borer (ACB), *Ostrinia furnacalis*, in open-top chambers were studied. The plants were infested with ACB and exposed to ambient and elevated (550 and 750 μl/l) CO_2_. E-CO_2_ increased the plant height and kernel number per ear. The plants had lower nitrogen contents and higher TNC: N ratios under E-CO_2_ than at ambient CO_2_. The response of plant height to E-CO_2_ was significantly dampened in plants with ACB infestation. However, the weight gain of the survivors declined in plants grown under E-CO_2_. Moreover, the plant damage caused by ACB was not different among the treatments. Overwintering larvae developed under E-CO_2_ had a lower supercooling point than those developed under ambient CO_2_. The results indicated that there was a positive effect of E-CO_2_ on the accumulation of maize biomass, i.e., the “air-fertilizer” effect, which led to a nutritional deficiency in the plants. The fitness-related parameters of ACB were adversely affected by the CO_2_-mediated decreased in plant nutritional quality, and ACB might alter its food consumption to compensate for these changes. Larval damage to maize under E-CO_2_ appears to be offset by this “air-fertilizer” effect, with reductions in larval fitness.

Since the Industrial Revolution, the huge consumption of fossil fuels and the destruction of natural habitats by the activities of humans have led to a continuous increase in CO_2_ concentrations in recent decades. The ambient CO_2_ concentration is expected to double within the 21^st^ century, i.e., it will increase from the current level of ∼379 μl/l to 700 μl/l^1^.

Because of the “fertilization effect”^2^, most C_3_ plants increase the rates of photosynthesis and photosynthate production when exposed to elevated CO_2_ (E-CO_2_)^3^. The phenotype response of plants to E-CO_2_ is generally an increased rate of growth and biomass accumulation^4^,^5^. However, not all plant species respond identically to E-CO_2_. Exposure to E-CO_2_ increased the biomass of *Agrostis capillaris* (L.) and *Poa alpine* (L.), but the biomass decreased in *Festuca vivipara* (L.)^6^. Additionally, the responses of plants may be different in the short term compared with the long term. The biomass of alfalfa, *Medicago sativa* (L.), and orchard grass, *Dactylis glomerata* (L.), was unaffected in the final two years of a 3-year experiment^7^. From the fertilization effect caused by the E-CO_2_, the increased rate of photosynthesis increases plant metabolism and generates plants with higher carbon-to-nitrogen ratios (C: N)^8^. Simultaneously, E-CO_2_ can lead to changes in plant secondary metabolism because of alterations in the plant allocation to defence chemistry and chemical signalling^9^. The concentrations of phenolics, terpenoids, condensed tannins, and gossypol were higher in Bt cotton, *Gossypium hirsutum* (L.), plants grown under E-CO_2_, but the concentration of the Bt toxin protein decreased^10^. In soybean, *Glycine max* (L.), plants grown under E-CO_2_ exhibited increased concentrations of quercet in the glycosides^11^. The plant response induced by the feeding of insect herbivores may be weaker, when grown are grown under E-CO_2_^12^. Depending on the species, the herbivore-induced response of plants may vary in different growth environments^13^.

Because of the elevated CO_2_-mediated dilution of the N content in plants results in a nutritional deficiency for protein-limited insect pests^14^, many insects compensate for the changes in plant quality by altering their food intake, which results in more severe damage or defoliation to the host plants^15^,^16^.

The C_3_ and C_4_ pathways of photosynthesis are distinct, and C_4_ plants are less sensitive to E-CO_2_ than C_3_ plants^17^,^18^. Therefore, an elevation in CO_2_ theoretically would not influence the rate of photosynthesis in C_4_ plants^19^, however, research has found that the rate of photosynthesis and the above ground biomass increased in C_4_ plants^20^,^21^,^22^. There are several explanations for this phenomenon^23^. A report showed that C_3_ grasses were more nutritious and had higher levels of proteins, nonstructural carbohydrates, and water and lower levels of fibre and toughness than C_4_ grasses under E-CO_2_^24^. Maize is a C_4_ plant and is the most important food and feed crop in China. The Asian corn borer (ACB), *Ostrinia furnacalis* (Guenée), is a key pest of maize and causes yield losses of 30% in various agro-climatic regions^25^. The ACB overwinters as fully developed larvae that are found in the maize stalks, cobs, and weed stems, or in a spun-silk covering in the plant debris^26^.

Numerous studies have been performed to predict the effects of rising CO_2_ concentrations on C_3_ crop-pest interactions in agriculture, but few studies have examined the effects on the C_4_ crops and their insect pests^27^. Similar to the C_3_ plants, E-CO_2_ also changes the interactions between maize and its pests. Yin *et al.* (2010)^16^ reported that the growth, development and consumption of *Helicoverpa armigera* (Hbn.) changed when it was fed maize grains grown under E-CO_2_. The exposure of maize plants and *Chilo partellus* (Swinhoe) to E-CO_2_ levels not only affected the growth and yield of maize, but also affected the development of the insect in the open-top chambers^28^.

The cold hardiness of an insect species is measured by its supercooling point (SCP), which influences the density of the overwintering population^29^. The SCP is influenced by various factors, including the host plant species and nutritional quality and the contents of water and of the cryoprotective substances in the larval body^30^,^31^,^32^,^33^. An understanding of the effects of E-CO_2_ on the SCP could provide direct evidence for the cold hardiness of the overwintering ACB populations in environments with future climate change.

The objectives of this study were to examine the effects of E-CO_2_ on the development and abundance of the 1^st^- and 2^nd^-generation ACBs and on the damage caused by the ACBs to the maize plants grown in open-top chambers. Additionally, the effect of E-CO_2_ on the cold hardiness of overwintering larvae was evaluated with tests to determine the supercooling points of diapause larvae. The information in this study on the performance and abundance of insects on plants (insect-plant interactions) under elevated levels of CO_2_ is as important as understanding the changes in herbivorous damage to agricultural commodities caused by global climate change.

## Results

### Maize plant chemistry

A negative effect of E-CO_2_ on the N content in maize plants was observed in the experiment (Table 1). Compared with the maize plants grown under ambient CO_2_, the N content significantly decreased by 8.0% and 17.0% for leaves and stalks, respectively, in maize plant grown under 750 μl/l CO_2_ (Table 1). The maize plants grown under 750 μl/l CO_2_ also had a 4.5% decrease in the N content of leaves compared with the maize plants grown under 550 μl/l CO_2_. There were no significant differences between the ambient and the 550 μl/l CO_2_ levels for the N content in leaves or stalks. A positive effect of E-CO_2_ on the C content of the maize plants was observed in the experiment (Table 1). The response of total non-structural carbohydrates (TNCs) including soluble sugars and starch, was consistent, with a significant effect of E-CO_2_ found only in the 750 μl/l CO_2_ treatment, for which the increasing was approximately 15.7% for soluble sugars and 8.3% for starch in leaves and 14.2% for soluble sugars and 11.9% for starch in stalks compared with maize plants grown under an ambient CO_2_ condition. The TNC: N ratio was significantly different among the treatments (Table 1). Compared with the ambient CO_2_ condition, the TNC: N ratio increased by 8.5 and 18.5% in leaves and 16.6% and 35.3% in stalks under E-CO_2_ levels (550 and 750 μl/l, respectively). Although the water content declined in the plants grown under elevated levels of CO_2_, the treatments were not significantly different (Table 1).

### Fitness of ACB larvae

For the 1^st^ generation of ACB, the larval survival among the CO_2_ treatments was not affected (Table 2). However for the 2^nd^-generation, the survival of the larvae decreased by 16.5% in 2012 and 21.0% in 2013 in the plant grown under elevated CO_2_ compared with the maize plants grown under ambient CO_2_. The average weight gain per larva (2^nd^ generatrion, diapause) declined significantly by 13.0% and 16.1%, respectively, when the larvae were fed maize plants grown under the two elevated levels of CO_2_ (550 and 750 μl/l), compared with the ambient CO_2_ (Table 3). Additionally, the cold hardiness of the diapause larvae was significantly affected by the CO_2_ concentrations. The average SCP of the diapause larvae that developed on the maize plants grown under E-CO_2_ (750 μl/l) was slightly lower (approximately 0.31 °C lower) than that for the larvae that developed on the maize plants grown under ambient CO_2_.

The fecundity of the moths that developed from the overwintering larvae was marginally affected by the CO_2_ levels (Table 3). Although the number of eggs laid by the females that developed from the overwintering larvae declined as the atmospheric CO_2_ concentration increased, the difference was not significant among the treatments.

### Maize plant growth and ACB damage ratings

**Figure 1:**
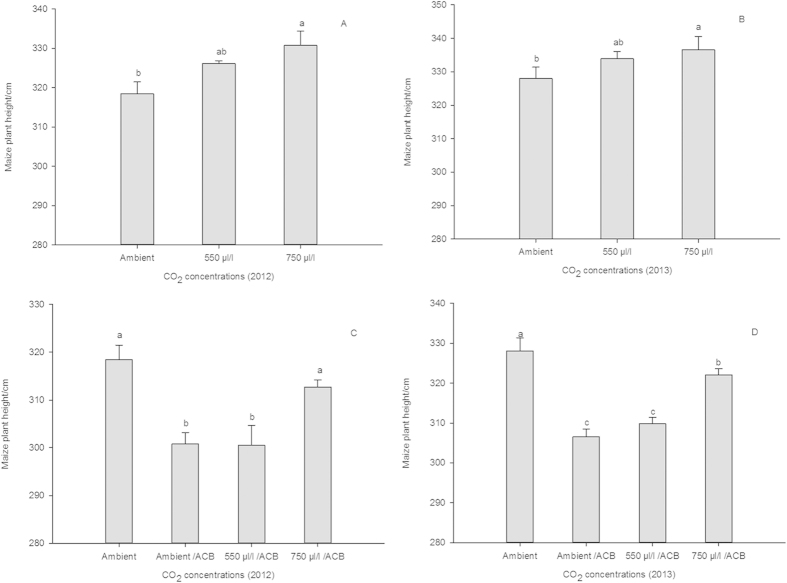
Height of maize plants in response to the effect of *Ostrinia furnacalis* infestation under different CO_2_ levels in 2012 and 2013. Different letters indicate significant differences among treatments (CONTRASTS test, P < 0.05).

The results revealed that the E-CO_2_ had a positive effect on the growth of plants, whereas the ACB infestation affected plant growth negatively (Fig. 1). A significant “air fertilizer” effect of E-CO_2_ was observed on maize plant growth (2012: F_2,6_ = 4.92, P < 0.05, Fig. 1A; 2013: F_2,6_ = 5.23, P < 0.05, Fig. 1B). Averaged over 2 years, the height of maize plants significantly increased by 3.85% under 750 μl/l compared with the ambient CO_2_. By contrast, the ACB damage at the whorl stage significantly suppressed the growth of maize plants (2012: F_2,8_ = 8.94, P < 0.01, Fig. 1C; 2013: F_2,8_ = 33.25, P < 0.01, Fig. 1D). Averaged over 2 years, the height of maize plants decreased by 6.54% with an ACB infestation at the whorl stage compared with the control. Overall, the ACB damage at the whorl stage completely dampened the positive height response of the maize plants to the E-CO_2_ (Fig. 1), which suggested that the larvae altered their food consumption to compensate for the changes in the quality of the maize plants. Additionally, the grain yields of the maize plants were significantly affected by the concentration of CO_2_ (Table 4). The average number of kernels per ear increased by 4.3% and 4.5% in 2012 and 2013, respectively, under E-CO_2_ (750 μl/l) compared with the ambient CO_2_. There was no significant difference in the weight per 100-kernels among the treatments. These results demonstrated the positive effect of E-CO_2_ on maize grain production. The damage ratings of the ACB were unaffected by the concentration of CO_2_ during the two years (Table 5). There were no significant differences in the number of tunnels per plant or in the length of cavities per plant among the treatments.

## Discussion

The effects of E-CO_2_ on maize plants have been assessed in a number of places with variable conditions. A few studies have suggested that maize plant are insensitive or less sensitive to elevated levels of CO_2_ in the absence of drought and heat^34^,^35^. Moreover, most studies have revealed that E-CO_2_ has a positive effect on the maize plant, i.e., maize plant are likely to have greater rates of photosynthesis and above ground biomass accumulation in addition to reduced transpirational water losses and increased water-use efficiencies^36^,^37^,^38^,^39^. The number of seed is also greater with E-CO_2_ than that of plants grown under ambient CO_2_ level (+5.0%)^40^. The stimulation of the photosynthesis and growth of maize plants under E-CO_2_ typically results in a reduction in leaf N content, or an increased in the TNC: protein ratio of maize grains^16^. The results of the present study were in accordance with previous studies that showed positive effects of E-CO_2_ on plant biomass, i.e. the heights of plant and the kernels per ear increased in the E-CO_2_ environment. Additionally, the chemical changes in the maize plants followed the general pattern of plants in responses to E-CO_2_, such as a decrease in the nitrogen content and an increase in the total nonstructural carbohydrates^41^. The TNC: N ratio significantly increased in response to E-CO_2_ in the present study, which suggested that the nutritional quality of the maize plant was reduced when the maize was grown under an E-CO_2_ condition. Similar research found that C_4_ grasses were poor host plants primarily because of their lower level of nutrient, higher level of fibre and greater toughness^42^.

Despite the direct response of many insects to E-CO_2_, the changes in the performance of herbivorous insects are intimately correlated with changes in the quality of food plants grown under E-CO_2_ conditions. The nitrogen (protein) content of the host plant is only one limiting nutrient for insect herbivores^43^, and a number of chemical compositions affect the nutritional quality of host plants^44^. A decrease in the foliar nitrogen content of host plants can affect the rates of development and survival of insect herbivores^45^. Moreover, insects that can compensate for the CO_2_-mediated dilution of foliar nitrogen by increasing the rate of feeding will experience retarded growth and will be subject to predation for a longer period of time (the slow-growth-high-mortality hypothesis)^46^. In present study, the survival decreased and the average weight gain per larva declined significantly when the larvae fed on the maize plants grown under E-CO_2_, possibly because of the CO_2_-mediated declines in the nitrogen content of the maize plants. These results indicated that the ACB is a protein-limited insect; the development and survival of the larvae were adversely affected by the CO_2_-mediated reduced suitability and nutritional quality of the host maize plants. Prominent among the many factors that affect the amount of plant tissues consumed by insect herbivores is that of the suitability and nutritional quality of their host plants. Studies have found that some leaf-chewing herbivores perform compensatory feeding and by increase the intake of foliage with a lower nitrogen content to meet their nutritional requirements under an E-CO_2_ environment^27^,^45^. As a consequence, levels of damage or defoliation increase. By contrast, the plants may be damaged less and have more undamaged foliar area when the E-CO_2_ causes an increase in plant biomass, and reduces the plant fitness-mediated population density of insect herbivores^47^. In the present study, the survival of the ACB larvae declined in the maize plants exposed to E-CO_2_ compared with the ambient treatment level. The E-CO_2_ also reduced the suppression of maize plant height caused by the ACB infestation. Therefore, the damage caused by the ACB to maize will be offset by the “air-fertilizer” effect for the plant and the reduced fitness of the insect herbivores on the host plants.

The ACB overwinters as fully developed larvae in maize stalks in northern China. The overwintering larval population (2^nd^-generation) has an important role in the overwinter survival of the ACB, and in the regulation of the population for the subsequent year^48^. The SCP of diapause larvae is largely related to the larval cold hardiness. In the present study, the diapause larvae that developed on the maize plants grown under 750 μl/l CO_2_ weighed less and had a slightly lower SCP than the larvae that developed on the maize plants grown under ambient CO_2_. These results suggested that there was a positive effect of E-CO_2_ on the cold hardiness of diapausing larvae, and many reports have shown that the host plants play a pivotal role in the coldhardiness of insect herbivore. For example, the average SCP was significantly lower for the 3^rd^ instar larvae of beet armyworm, *Spodoptera exigua* (Hbn.), that developed from cabbage than those that developed from pakehoi, shallot and spinach^49^. Similar evidence was found for the hemlock looper, *Lambdina fiscellaria* (Guenée)^50^. The host plant quality affected the overwintering success of the leaf beetle, *Chrysomela lapponica* (L.)^32^, the hypothesis to explain this result was that the high water content in the high-weight beetles of *C. lapponica* might be the primary cause of the increased winter mortality^51^. In the present study, the larval body weight was lower and the increase in the SCP occurred under E-CO_2_ conditions; however, these results might also be associated with the nutritional quality and the lower water content of the maize plants that were grown under E-CO_2_ than those that were grown in ambient conditions.

In this study, the survival rate of the overwintering larvae (data not shown) was not influenced by the E-CO_2_ although there was a small decrease in the SCPs of the larvae. Additionally, the number of eggs laid per female that developed from the overwintering larvae declined at E-CO_2_ treatments although the difference was not statistically significant compared with the ambient condition. Taken together, there was insufficient evidence to conclude that the ACB exhibited a direct response to the elevated levels of CO_2_.

## Methods

### Open-top chambers

This experiment was conducted in regular octagonal open-top chambers (4.20 m in diameter by 3.0 m in height) located at the Gongzhuling Experimental Station of the Institute of Plant Protection, Chinese Academy of Agricultural Sciences, Gongzhuling, Jilin Province, China (43°30′ N, 124°47′ E; 224.9 m above sea level). Three levels of CO_2_ were applied continuously, i.e., ambient CO_2_ (∼390 μl/l) and E-CO_2_ (550 μl/l and 750 μl/l), which represented the current and predicted levels of CO_2_ in future years, respectively^52^,^53^, each treatment was replicated four times, for a total of twelve chambers in the experiment. The air was continuously distributed from the blowers into the chambers through a water curtain cooling system into perforated polyethylene ducts inside the chamber base-wall at 10 cm above the level of the soil. The CO_2_ was added to the inlet airstreams in the chambers of the elevated treatments to reach the target CO_2_ concentrations (550 μl/l and 750 μl/l). The concentrations were monitored and adjusted with a CO_2_ sensor (JQAW-8VACD, ColliHigh Company, Beijing, China) once every 60 s to ensure relatively stable levels of CO_2_. The actual mean CO_2_ concentrations in the chambers were 542 ± 14 μl/l and 746 ± 15 μl/l for the two E-CO_2_ levels, whereas in the ambient chambers, the concentration was ∼390 μl/l. The concentrations in the ambient chambers were monitored but were not controlled. The automatic-control system for adjusting the CO_2_ concentration was similar to that described by Chen *et al.* (2005)^54^, which also included the specifications for the open-top chambers. The open tops of the OTCs were covered with nylon netting to prevent insect immigration. The detailed descriptions of the chamber design and the experimental set-up will be published separately.

### Maize variety and growth conditions

The maize (*Zea mays* L., Poaceae) (XY335, DuPont Pioneer Hi-Bred, Beijing, China) was planted with 50 cm row spacing (a total of 60 plants per chamber) in the open-top chambers on 10 May, 2012 and 15 May, 2013. This hybrid is typical of those grown for commercial production, and the field management was consistent with the common cultural practices used in local farming.

### Insect stocks and plant infestation

The ACB neonates used in this study were obtained from a laboratory colony that originated from a field population collected every year, which was maintained on a regular artificial diet for ACBs^26^ for 3–4 generations in the Institute of Plant Protection, Chinese Academy of Agricultural Science.

The plants were infested when they developed to the whorl and silking stages, which represent the 1^st^- and the 2^nd^-generation infestations in nature, respectively. Before the first infestation, each chamber was separated into two plots with screen to prevent the larvae from transferring between the two plots. The two plots in a chamber were used as treatments with either the 1^st^-generation infestation or the 2^nd^-generation infestation. Each plant was infested with 50 neonates (<24 h) of the ACB with traditional artificial infestation techniques similar to those described by He *et al.* (2000)^55^ on 29 and 28 June (the 1^st^-generation) and on 8 and 7August (the 2^nd^-generation) in 2012 and 2013. To avoid exposing the neonates to high temperature and direct sunlight, the infestations were applied during the late afternoon to evening. Nine chambers were used for the ACB infestation, which included three for each level of CO_2_, and another three chambers were used for the assays on chemical composition of the maize leaves.

### Larval survival sampling and maize plant damage rating

For the 1^st^-generation, when the fifth instar larvae of the ACB were observed (24 July, 2012 and 25 July, 2013) in the maize field around the open-top chambers, the maize plants were dissected, and the plant height and the number of larvae and tunnels and the tunnel lengths were recorded or measured. For the 2^nd^-generation, the maize plants were dissected on 22 September, 2012 and 20 September, 2013 (ready to harvest), the supercooling points and the overwinter status for 2012 were determined for the larvae from the dissected maize plants.

### Supercooling points and overwinter status

The larvae were collected from the dissected maize plants during the autumn harvest, introduced into plastic centrifuge tubes with an air hole punched through the bottom (1.5 ml) and placed into cartons maintained, in the open-top chambers during the winter. The supercooling points of the larvae after overwintering were determined on 18 January 2012. A tube was constructed by removing the bottom part from a micro-centrifuge tube (0.5 ml), which was used to position larvae that were connected to a multichannel temperature recorder (TMC-40A, designed by the Institute of Agro-meteorology, Chinese Academy of Agricultural Sciences, Beijing). These tubes were then placed in the temperature test chamber (Heraeus-Votsch VM 04/100) at 0 °C to equilibrate for 24 h before cooling at a rate of 1 °C/min until a temperature of −40 °C was reached. The lowest temperature reached before the release of the latent heat of fusion was recorded as the supercooling point (SCP). Twenty-four larvae were tested for each treatment, and each treatment was replicated three times. The larvae that were not unsupercooled with the same origin were then placed individually into modified 5-ml centrifuge tube (two holes 1 mm in diameter were made in each lid; sterilized) with a piece of wet cotton as a moisture source and a piece of corrugated paper as a cryptic habitat. Finally, these larvae were reared to pupation at 26 °C, 70% RH, and with a 16:8 h (L:D) photoperiod. The newly emerged moths from each treatment were transferred in pairs to an oviposition cage (11 cm × 8 cm × 8 cm), which was covered with a piece of waxed paper as an oviposition substrate^26^. The number of eggs laid per female was recorded daily.

### Chemical compositions of maize leaves

One entire unfolded ear leaf and stalk was collected from each maize plant in the OTCs for tissue samples for the chemical composition assays on the 75^th^ day after sowing. Ten maize plants were selected at random from each of the three CO_2_ treatments, on three separate occasions, for a total of 30 leaves and 30 stalks per treatment. The water content, as a proportion of fresh weight, was calculated after the maize leaves and stalks were dried at 80 °C for 72 h. The total non-structural carbohydrates (primarily soluble sugars and starch) were analysed using the method of Tissue and Wright (1995)^56^. The nitrogen content was assayed using a Kjeltec N analyser (Model KDY-9830; Foss automated Kjeltec instruments, Beijing, China).

### Data analyses

One-way analyses of variance (ANOVAs) were used to analyse the effects of elevated CO_2_ on the chemical compositions of maize leaves and stalks, larval survival per maize plant, larval body weights, supercooling points, kernel numbers, 100-kernel weights, maize plant heights, the ACB damage ratings, and the number of eggs. All data were analysed with a general linear model procedure (PROC GLM) (SAS Institute 2001). Differences in the treatments were compared using CONTRASTS test. The significance level was set at P < 0.05. Before the analyses, the data were subjected to standard transformations to improve their normality and the homogeneity of variance. The percentage data were arcsine transformed to meet the assumptions of homogeneity of variance.

## Additional Information

**How to cite this article**: Xie, H. *et al.* A field experiment with elevated atmospheric CO_2_-mediated changes to C_4_ crop-herbivore interactions. *Sci. Rep.* **5**, 13923; doi: 10.1038/srep13923 (2015).

## Figures and Tables

**Table 1:** Chemical composition (mean ± SE) of maize leaves and stalks (without *Ostrinia furnacalis* infestation) grown under different CO_2_ levels in 2012.

**Organs**	**CO_2_levels**	**Water**	**N**	**Soluble sugar**	**Starch**	**TNC: N**
	**(μl/l)**	**(%)**	**(%)**	**(mg/g)**	**(mg/g)**	
Leaves	Ambient	76.86 ± 2.25	3.26 ± 0.08a	27.94 ± 0.61a	118.74 ± 0.89a	4.49 ± 0.07a
	550	76.17 ± 1.67	3.14 ± 0.04a	29.94 ± 1.46ab	122.93 ± 1.89ab	4.87 ± 0.07b
	750	73.67 ± 1.96	3.00 ± 0.04b	32.34 ± 0.47b	128.61 ± 1.29b	5.32 ± 0.02c
	F_2,6_	0.69	5.33	7.98	4.93	58.85
	P	0.54	0.04	0.02	<0.04	<0.01
Stalks	Ambient	92.07 ± 1.16	1.06 ± 0.04a	94.57 ± 1.56a	84.57 ± 1.18a	17.01 ± 0.74a
	550	91.34 ± 1.04	0.94 ± 0.03ab	99.10 ± 1.39ab	88.78 ± 3.45ab	19.83 ± 0.94b
	750	91.75 ± 1.08	0.88 ± 0.03b	107.97 ± 3.40b	94.60 ± 0.48b	23.01 ± 1.02c
	F_2,6_	0.09	7.80	5.64	4.98	10.86
	P	0.91	0.02	0.04	0.04	0.01

Means within a column followed by different letters are significantly different (CONTRASTS test, P < 0.05). N: Nitrogen; TNC: Total non-structural carbohydrates.

**Table 2:** Survival of *Ostrinia furnacalis* on maize plants grown under different CO_2_ levels for two generations in 2012 and 2013.

**CO**_**2**_**levels**	**Generation 1**		**Generation 2**	
**(μl/l)**	**2012**	**2013**	**2012**	**2013**
Ambient	4.0 ± 0.6	2.8 ± 0.2	5.7 ± 0.4a	4.4 ± 0.3a
550	3.9 ± 0.6	2.6 ± 0.1	5.2 ± 0.3ab	3.8 ± 0.1b
750	3.9 ± 0.6	2.7 ± 0.7	4.8 ± 0.3b	3.5 ± 0.1c
F_2,6_	0.01	0.77	11.50	5.95
P	0.99	0.50	<0.01	0.04

Means within a column followed by different letters are significantly different (CONTRASTS test, P < 0.05).

**Table 3:** Body weight and supercooling points (SCP) (mean ± SE) for overwintering larvae and female fecundity of *Ostrinia furnacalis* from different CO_2_ levels in 2012.

CO_2_levels(μl/l)	Larval bodyweight	**Supercooling point**	**SCP range**	**Number of egg**
	**(mg)**	**(°C)**	**(°C)**	**/female**
Ambient	124.35 ± 2.78a	−24.77 ± 0.24a	−14.56 ∼ −28.50	248.68 ± 13.60
550	108.17 ± 1.77b	−25.03 ± 0.13ab	−14.55 ∼ −28.99	215.90 ± 14.81
750	104.29 ± 2.15b	−25.08 ± 0.81b	−15.67 ∼ −28.95	202.20 ± 10.12
F_2,6_	7.80	20.08		3.15
P	0.02	<0.01		0.05

Means within a column followed by different letters are significantly different (CONTRASTS test, P < 0.05).

**Table 4:** Effects of CO_2_ levels on yield of maize with *Ostrinia furnacalis* infestation at the silking stage in 2012 and 2013.

**CO_2_levels**	**Kernel number/ear**		**100-kernel weight (g)**	
**(μl/l)**	**2012**	**2013**	**2012**	**2013**
Ambient	413.4 ± 4.2b	431.6 ± 3.8b	35.08 ± 1.09	31.56 ± 1.31
550	418.2 ± 2.7b	434.5 ± 4.3b	34.44 ± 1.87	31.81 ± 0.29
750	431.3 ± 3.6a	450.9 ± 2.6a	35.32 ± 1.04	32.73 ± 0.67
F_2,6_	5.08	8.22	0.11	0.51
P	0.04	0.02	0.90	0.63

Means within a column followed by different letters are significantly different (CONTRASTS test, P < 0.05).

**Table 5:** Effects of CO_2_ levels on damage ratings of maize plants with the 1^st^ or 2^nd^ generation infestation of *Ostrinia furnacalis* in 2012 and 2013.

**CO_2_levels**	**Number of tunnels/plant**		**Length of tunnels (cm)**	
**(μl/l)**	**2012**	**2013**	**2012**	**2013**
**Generation 1**				
Ambient	3.1 ± 0.5	2.9 ± 0.3	7.36 ± 0.98	7.11 ± 0.53
550	3.7 ± 0.7	2.7 ± 0.3	8.14 ± 0.33	6.40 ± 0.08
750	4.0 ± 1.1	2.6 ± 0.4	8.94 ± 0.51	6.99 ± 0.38
F_2,6_	0.54	0.66	0.54	1.30
P	0.61	0.55	0.61	0.34
**Generation 2**				
Ambient	5.9 ± 0.4	4.2 ± 0.5	21.14 ± 4.43	19.28 ± 1.08
550	6.2 ± 0.6	3.1 ± 0.6	23.90 ± 4.23	18.90 ± 1.35
750	4.5 ± 0.5	4.1 ± 0.2	17.50 ± 5.07	17.72 ± 1.18
F_2,6_	0.39	0.28	1.09	4.93
P	0.69	0.77	0.39	0.06
